# *Helicobacter pylori* Diagnostic Tests Used in Europe: Results of over 34,000 Patients from the European Registry on *Helicobacter pylori* Management

**DOI:** 10.3390/jcm12134363

**Published:** 2023-06-28

**Authors:** Natalia García-Morales, Ángeles Pérez-Aísa, Giulia Fiorini, Bojan Tepes, Manuel Castro-Fernández, Alfredo Lucendo, Irina Voynovan, Luis Bujanda, Ana Garre, Luis Rodrigo, Samuel Jesús Martínez Domínguez, Maja Denkovski, Jose M. Huguet Malavés, Laimas Jonaitis, Renate Bumane, Oleg Zaytsev, Pilar Mata Romero, Jesús Barrio, Luis Fernández-Salazar, Aiman Silkanovna Sarsenbaeva, Inmaculada Ortiz Polo, Sergey Alekseenko, Ilaria Maria Saracino, Dino Vaira, Alma Keco-Huerga, Dmitry Bordin, Antonio Gasbarrini, Frode Lerang, Theodore Rokkas, Juozas Kupčinskas, Marcis Leja, Gulustan Babayeva, Ricardo Marcos Pinto, Ante Tonkić, Sinead Smith, Perminder Phull, Gyorgy M. Buzas, Halis Simsek, Doron Boltin, Oleksiy Gridnyev, Marino Venerito, Vladimir Milivojevic, Núria Torà, Anna Cano-Català, Leticia Moreira, Olga P. Nyssen, Francis Mégraud, Colm O’Morain, Javier P. Gisbert, Ignasi Puig, on behalf of Hp-EuReg Investigators

**Affiliations:** 1 Digestive Service, Complexo Hospitalario Universitario de Vigo, Sergas, South Galicia Health Research Institute, 36312 Vigo, Spain; natgmorales@gmail.com; 2 Unidad de Digestivo, Hospital Costa del Sol Marbella, Redes de Investigación Cooperativa Orientada a Resultados en Salud (RICORS), 29603 Marbella, Spain; drapereza@hotmail.com; 3 Department of Surgical and Medical Sciences, IRCCS AOU S. Orsola, 39015 Bologna, Italy; giulia.fiorini@aosp.bo.it (G.F.); saracinoilariamaria@gmail.com (I.M.S.); berardino.vaira@unibo.it (D.V.); 4 AM DC Rogaska, 3250 Rogaska Slatina, Slovenia; bojan.tepes@siol.net; 5 Hospital Valme, 41014 Sevilla, Spain; mcastrof1955@gmail.com (M.C.-F.); almakh94@hotmail.com (A.K.-H.); 6 Hospital de Tomelloso, 13700 Ciudad Real, Spain; ajlucendo@hotmail.com; 7 A.S. Loginov, Clinical Scientific Centre, 111123 Moscow, Russia; irinavmgd2@mail.ru; 8 Department of Gastroenterology, Biodonostia Health Research Institute, Centro de Investigación Biomédica en Red de Enfermedades Hepáticas y Digestivas (CIBERehd), Universidad del País Vasco (UPV/EHU), 20014 San Sebastián, Spain; luis.bujanda@osakidetza.net; 9 Hospital Universitario de La Princesa, Instituto de Investigación Sanitaria Princesa (IIS-Princesa), Universidad Autónoma de Madrid (UAM) and Centro de Investigación Biomédica en Red de Enfermedades Hepáticas y Digestivas (CIBERehd), 28006 Madrid, Spain; anagarre.laprincesa@gmail.com (A.G.); javier.p.gisbert@gmail.com (J.P.G.); 10 Hospital Central de Asturias, 33011 Oviedo, Spain; lrodrigosaez@gmail.com; 11 Hospital Clínico Lozano Blesa, Centro de Investigación Biomédica en Red de Enfermedades Hepáticas y Digestivas (CIBERehd), 50009 Zaragoza, Spain; samuelmartinez94@hotmail.com; 12 Diagnostic Center Bled, 4260 Bled, Slovenia; maja.denkovski@gmail.com; 13 Hospital General Universitario de Valencia, 46014 Valencia, Spain; josemahuguet@gmail.com; 14 Department of Gastroenterology, Lithuanian University of Health Sciences, 44307 Kaunas, Lithuania; laimasjonaitis@yahoo.com (L.J.); juozas.kupcinskas@lsmuni.lt (J.K.); 15 Digestive Diseases Centre GASTRO, LV-1006 Riga, Latvia; renate.bumane@gmail.com (R.B.); cei@latnet.lv (M.L.); 16 First Clinical Medical Centre, 601900 Kovrov, Russia; locot@yandex.ru; 17 Hospital Universitario de Cáceres, 10004 Cáceres, Spain; pmataromero@gmail.com; 18 Hospital Rio Hortega, 47012 Valladolid, Spain; jbarrioa95@gmail.com; 19 Hospital Clínico de Valladolid, 47003 Valladolid, Spain; luisfernsal@gmail.com; 20 Gastroenterologist Department of Regional Clinical Hospital N3, 454080 Chelyabinsk, Russia; aiman-ss@yandex.ru; 21 Hospital la Fe, 46026 Valencia, Spain; ortizpolo.inmaculada@gmail.com; 22 Far Eastern State Medical University, 680000 Khabarovsk, Russia; sa.alexeenko@gmail.com; 23 Gastroenterology Unit, A.S. Loginov Moscow Clinical Scientific Center, 111123 Moscow, Russia; dbordin@mail.ru; 24 Gastroenterology Unit, Department of Outpatient Therapy and Family Medicine, Tver State Medical University, 170100 Tver, Russia; 25 Gastroenterology Unit, Department of Propaedeutic of Internal and Gastroenterology, A.I. Yevdokimov Moscow State University of Medicine and Dentistry, 127473 Moscow, Russia; 26 Medicina Interna e Gastroenterologia, Fondazione Policlinico Universitario A. Gemelli IRCCS, Università Cattolica del Sacro Cuore, 00168 Roma, Italy; antonio.gasbarrini@unicatt.it; 27 Central Hospital of Ostfold, 1601 Fredrikstad, Norway; flerang@online.no; 28 Henry Dunant Hospital, 115 26 Athens, Greece; sakkor@otenet.gr; 29 Memorial Klinika, 1096 Baku, Azerbaijan; ghbabayeva@gmail.com; 30 Centro Hospitalario do Porto, 4050-101 Porto, Portugal; ricardomarcospinto@sapo.pt; 31 Department Gastroenterol & Hepatol, University Hospital Centre Split, 2100 Split, Croatia; ante.tonkic@gmail.com; 32 Tallaght Hospital, DR24 NR0A Dublin, Ireland; smithsi@tcd.ie; 33 Aberdeen Royal Infirmary, Aberdeen AB25 2ZN, UK; p.s.phull@abdn.ac.uk; 34 Gastroenterology, Ferencváros Health Centre, Mester utca 45, 1095 Budapest, Hungary; drbgym@gmail.com; 35 Internal Med Gastroenterol Department, Hacettepe University School of Medicine, 06230 Ankara, Turkey; hcsaglik@gmail.com; 36 Division of Gastroenterology, Rabin Medical Center, Sackler School of Medicine, Tel Aviv University, Tel Aviv 4941492, Israel; dboltin@gmail.com; 37 L.T. Malaya Therapy National Institute of the National Academy of Medical Sciences, ID 70483 Kharkiv, Ukraine; alex.gridnyev@gmail.com; 38 Department of Gastroenterology, Hepatology and Infectious Diseases, Otto-von-Guericke University Hospital, 44, 39120 Magdeburg, Germany; m.venerito@med.ovgu.de; 39 Clinical Center of Serbia, 11000 Belgrade, Serbia; dotorevlada@gmail.com; 40 GOES Research Group, Unitat de Recerca i Innovació, Athaia Xarxa Assistencial Universitària de Manresa, 08243 Manresa, Spain; ntora@althaia.cat (N.T.); acano@aegastro.es (A.C.-C.); 41 Hospital Clínic de Barcelona, Centro de Investigación Biomédica en Red de Enfermedades Hepáticas y Digestivas (CIBERed), 08036 Barcelona, Spain; lmoreira@clinic.cat; 42 INSERM U1312, Université de Bordeaux, 33000 Bordeaux, France; francis.megraud@u-bordeaux.fr; 43 Rabin Medical Center, Beilinson Campus, Petah Tikva 49100, Israel; colmomorain@gmail.com; 44 Althaia Xarxa Assistencial Universitària de Manresa and Universitat de Vic-Universitat Central de Catalunya (UVicUCC), 08242 Manresa, Spain; ignasipuig@gmail.com

**Keywords:** diagnostic tests, gastrointestinal endoscopy, *Helicobacter pylori*, histology, urea breath test

## Abstract

Background and aims: Several methods are available to diagnose *Helicobacter pylori* infection. Our objective was to evaluate the tests used for both the initial diagnosis and the confirmation of eradication after treatment in Europe. Methods: The European Registry on the management of *Helicobacter pylori* infection is an international, multicentre, prospective, non-interventional registry aiming to evaluate the management of *Helicobacter pylori*-infected patients in Europe. Countries with at least 100 cases registered from June 2013 to April 2021, and with a validated diagnostic method were analysed. Data were quality reviewed. Results: A total of 34,920 adult patients from 20 countries were included (mean age 51 years; 61% women). To establish the initial diagnosis, invasive tests were performed in 19,801 (71%) patients, non-invasive in 11,369 (41%), and both in 3437 (12%). The most frequent were histology (n = 11,885; 43%), a rapid urease test (n = 10,636; 38%) and an urea breath test (n = 7577; 27%). According to the age, invasive tests were indicated in 11,179 (77%) ≥50 years, and in 8603 (65%) <50 years. Depending on the country, the use of invasive tests ranged from 29–99% in <50 years to 60–99% in ≥50. Most of the tests used to confirm eradication were non-invasive (n = 32,540; 93%), with the urea breath test being the most frequent (n = 32,540; 78%). In 2983 (9%) post-treatment tests, histology (n = 1887; 5%) or a rapid urease test (n = 1223; 4%) were performed. Conclusion: A great heterogeneity was observed for the initial diagnosis and confirmation of the eradication. The reasons for the apparent lack of adherence to the clinical guidelines should be further explored.

## 1. Introduction

Approximately half of the population worldwide is infected by *Helicobacter pylori (H. pylori)* [1,2]. Its prevalence varies according to geographic areas, as it is influenced by different factors such as age, socioeconomic status and hygienic conditions [3]. Patients with *H. pylori* infection are at risk of developing various complications, mainly gastroduodenal ulcer, gastric adenocarcinoma and lymphoma [4]. Thus, a proper diagnosis followed by an effective treatment, and a confirmation of bacterial eradication, is especially important for the clinical outcome and prognosis of these patients [5].

Several tests are indicated to establish the initial diagnosis of *H. pylori* infection [6]. The urea breath test (UBT) is considered the most accurate non-invasive test for its high sensitivity and specificity [7]. When UBT is not available, monoclonal stool antigen tests (MSAT) are also a valid alternative [8]. Serology tests are generally not recommended, except if a local validation has been performed. Other tests such as rapid (“office”) serological or saliva tests are not recommended in this scenario [6,9].

Invasive tests are performed by obtaining tissue samples collected with upper gastrointestinal endoscopy. Rapid urease test (RUT) is the first-line diagnostic test. Histology is also recommended as it allows us to assess gastritis and precancerous lesions if suspected [6]. Regarding culture, the generalised use of susceptibility-guided therapy for *H. pylori* treatment in routine clinical practice, either as a first-line or as a rescue treatment, is not performed due to the low cost-effectiveness of culture and questionable clinical efficacy compared to empirical highly effective quadruple therapies [10,11,12].

The type of test used depends on the characteristics of the patient and the presence or absence of red flags. The “test and treat” strategy is based on the investigation of the presence of *H. pylori* and its subsequent eradication when detected in young (<50 years old) patients with dyspeptic symptoms and the absence of alarm symptoms [13]. However, in the case of alarm symptoms or age over 50, an upper gastrointestinal endoscopy should be performed in order to rule out gastric cancer or other organic pathologies [13,14].

Furthermore, once the diagnosis of *H. pylori* infection has been established and antibiotic treatment prescribed, a confirmatory eradication test should be performed. UBT is generally the test of choice to confirm eradication, but MSAT may be a valid alternative. Serology should not be used to confirm eradication due to its lack of efficacy, and the use of invasive tests is also generally not necessary [10].

Taking into account all these considerations, and since no information is currently available about clinical practice regarding the diagnostic process of *H. pylori*, the present study aims to evaluate the type of tests used in Europe for the initial diagnosis of *H. pylori* infection as well as for the control of eradication after treatment. The aim of the current study was to evaluate the type of tests used pre- and post-treatment in first-line treatment, to assess the type of control tests used to confirm the eradication of the infection both in treatment-*naïve* and rescue treatment patients, and ultimately to evaluate the evolution in the use of these methods in Europe.

## 2. Methods

### 2.1. European Registry on H. pylori Management

The “European Registry on *Helicobacter pylori* management” (Hp-EuReg) brings together information on the real clinical practice of most European countries, including thousands of patients [15]. It represents a good mapping overview of the current situation regarding the diagnostic management of *H. pylori*, allowing not only for the continuous assessment of the implementation of clinical recommendations agreed on medical consensus, but also of the possible strategies for improvement. The general aim of the Hp-EuReg was to set up an ongoing database in which a large representative sample of European gastroenterologists would systematically record their routine management of patients infected with *H. pylori* [15].

This analysis focused on the Hp-EuReg, an international, multicentre, prospective, non-interventional registry that started in 2013 and was promoted by the European Helicobacter and Microbiota Study Group (www.helicobacter.org accessed on 20 June 2023).

At the moment of the analysis, 27 countries were participating. Criteria for country selection, national coordinators and recruiting gastroenterologists and investigators are detailed in the published protocol [15]. Cases were managed and registered according to their routine clinical practice. The Hp-EuReg protocol was approved by the Ethics Committee of La Princesa University Hospital (Madrid, Spain), which acted as reference Institutional Review Board; was classified by the Spanish Drug and Health Product Agency and registered at ClinicalTrials.gov under the code NCT02328131. Written informed consent was obtained from each patient included in the study.

Data were recorded in an electronic case report form using the REDCap collaborative platform hosted at “Asociación Española de Gastroenterología” (AEG, www.aegastro.es accessed on 20 June 2023), a non-profit scientific and medical society focused on gastroenterology research [16,17].

### 2.2. Patients

All *H. pylori*-infected adult patients evaluated by a gastroenterologist were collected from June 2013 to April 2021. All cases with information regarding the tests used to establish the initial diagnosis and the confirmation of the eradication were included, including both treatment-*naïve* patients as well as further subsequent eradication treatments’ attempts. For the purpose of this analysis, a threshold of at least 100 complete records by country was established to avoid non-representative geographical areas (i.e., with a small sample size), encompassing a total of 20 different countries.

### 2.3. Data Management

The variables analysed included: patient’s characteristics such as age, gender and ethnicity, country of origin, line of treatment, and tests used for diagnosis, and confirmation of eradication. Histology, RUT, culture, or biochemical methods, such as polymerase chain reaction (PCR) or fluorescence in situ hybridization (FISH), were considered as invasive tests, and UBT, serology, monoclonal, and polyclonal stool antigen tests as non-invasive.

Data were subjected to monitoring (per country and centre) and were quality-checked to ensure coherence and data reliability. Sub-analyses were conducted depending on a patient’s age, country, and line of treatment, whenever possible. For the bivariate analyses, we selected those patients for whom only one type of test (invasive or non-invasive) was indicated for the initial diagnosis.

### 2.4. Statistical Analyses

Continuous variables were summarised using means and standard deviation (SDs) for normal distributions, and medians with the interquartile range for non-normal distributions. Categorical variables were summarised using absolute values together with their relative frequencies (%) and their corresponding 95% confidence intervals (CI).

The Chi-square test was used to compare categorical variables or Fisher’s exact test in contingency tables when expected frequencies were less than five. The Mann–Whitney U test was used for non-parametric variables comparisons.

Logistic regression was conducted to evaluate the association between the type of test indicated for the initial diagnosis and the patient’s characteristics. Unadjusted odds ratios (OR) and 95% CIs were reported.

The evolution in the use of diagnostic tests between 2013 and 2021 was also analysed.

In all analyses, a two-sided α-level of 0.05 was considered statistically significant.

## 3. Results

### 3.1. Patients’ Characteristics

**Figure 1:**
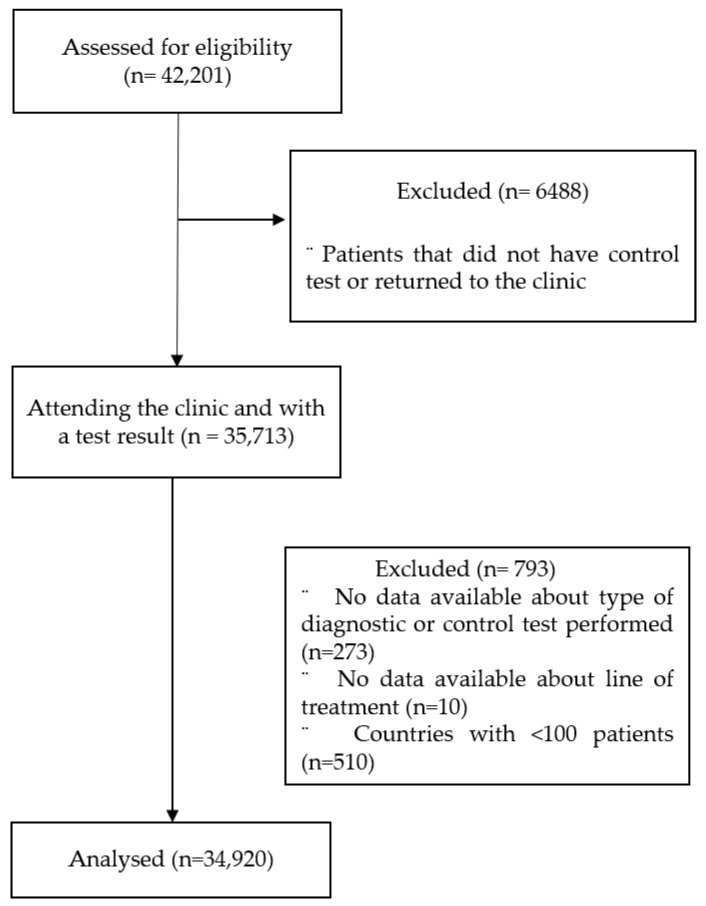
Study flow diagram.

By April 2021, 34,920 patients from 20 countries were included in the analysis. Patients’ flow-diagram is shown in Figure 1.

The patients’ mean age was 51 years (SD ± 14) and most were women (n = 21,350, 61%) and Caucasian (n = 31,058, 89%). Further patients’ characteristics are shown in Table 1.

Patients from Spain, Russia, and Italy represented the majority of the data (74%) evaluated. The participation by country including more than 100 patients is shown in Table S1.

### 3.2. Initial Diagnosis in Treatment-naïve Patients

To establish the initial diagnosis of *H. pylori* infection in treatment-*naïve* patients, non-invasive tests only were performed in 29% (95% CI 0.28–0.29) of cases, invasive tests only in 59% (95% CI 0.58–0.60) and both types of tests in 12% (95% CI 0.11–0.12). The most frequently used diagnostic tests were: histology (43%), RUT (38%), and UBT (28%). Further details are shown in Table 2.

When the invasive tests were analysed, histology was reported as the unique test in 30% of patients and RUT in 26% (Table S2). The proportion of invasive and non-invasive tests differed widely according to the country (Table S3).

In accordance with the age of the patient, an invasive test was used in 77% of those ≥50 years, and in 65% of those <50 years old.

Data by country showed that invasive testing in those patients <50 years ranged between 29% and 99% of cases and between 60% and 99.5% in those ≥50 years (Table 3). There were no significant differences in the proportion of invasive tests used according to age in nine out of the 20 evaluated countries.

In the comparative univariate analyses, and therefore after excluding those patients in whom both an invasive and non-invasive test were performed, the use of an invasive test was significantly associated with the following factors: age ≥ 50 years (74% vs. 60%; OR 1.8, 95% CI 1.7–1.9, *p* < 0.001), male gender (70% vs. 66%; OR 1.2 95% CI 1.2–1.3, *p* < 0.001), and country of origin (*p* < 0.01) (Table 4).

### 3.3. Evolution of the Initial Diagnostic Tests Used in Treatment-naïve Patients

Between 2013 and 2021, the use of ^13^C-UBT to diagnose the *H. pylori* infection ranged from 20 to 28% as a minimum and maximum rate over the years, MSAT from 4 to 9%, histology from 35 to 52% and RUT from 18 to 54%. The evolution in the proportions of the type of test used for the initial diagnosis throughout the years in Europe is shown in Table S4. Sub-analyses performed by country (with more than 1000 patients) are shown in Table S5.

### 3.4. Control Tests to Confirm the Eradication in Treatment-naïve and Rescue Treatment Patients

The type of tests used to evaluate the eradication of the bacterial infection were most frequently non-invasive (93%; 95% CI 0.92–0.93), both after the first-line treatment and after rescue therapies, mainly by means of UBT (78%). In 8.5% (95% CI 0.08–0.09) of the cases, eradication was assessed with an invasive test requiring upper gastrointestinal endoscopy and biopsies for histology in 5.4% and/or RUT in 3.5%.

Data on the control tests used both after first-line and rescue treatments are shown in Table 5.

Note that histology was conducted in 4.9%, RUT in 3.1% and both tests in 0.6% to confirm eradication after the first-line treatment (Table S6). Further details of the distribution of the type of tests indicated by country are shown in Table S7.

### 3.5. Evolution of the Control Tests

Between 2013 and 2021, the most frequently used tests to confirm the eradication were: ^13^C-UBT (minimum and maximum rate of use over the years ranging from 67 to 86%), followed by MSAT (6–21%) and RUT (1–4%). The evolution of the type of test used for the confirmation of the eradication throughout the years in Europe is shown in Table S8. Sub-analyses performed by country (with more than 1000 patients) are shown in Table S9.

### 3.6. Use of Culture in Treatment-naïve and Rescue Treatment Patients

Overall, culture was performed in 11% of cases. Specifically in treatment-*naïve* patients, culture testing was conducted in 10.5%, in 15% of patients receiving a rescue treatment (11% in second-line treatment and 24% in the remaining rescue treatment lines) (Table S10).

## 4. Discussion

This is the largest and first study to our knowledge evaluating the tests used for the diagnosis and control of the eradication treatment in the management of *H. pylori* infection. The results were obtained by analysing data from over 35,000 patients from 20 different European countries.

Our study showed: (1) a great heterogeneity among European countries in the use of invasive and non-invasive tests for the initial diagnosis of *H. pylori* infection; (2) invasive tests for the initial diagnosis of *H. pylori* infection were performed probably unnecessarily in the majority of patients, mainly in those <50 years old; (3) a non-negligible number of patients ≥50 years old were only tested with non-invasive tests (that is, without endoscopy); (4) culture was performed in a relatively small number of patients both in first-line and rescue-treatment patients; (5) UBT was by far the most common test used to confirm the eradication of *H. pylori* infection; however, invasive tests were still used in a low proportion.

The present study is the first to analyse and perform a mapping review of the diagnostic methods used to detect the *H. pylori* infection in Europe. The results demonstrated that there is a great heterogeneity between the different European countries when choosing the test for both the initial diagnosis and the control of the *H. pylori* eradication. These findings suggest that the established recommendations for the correct diagnosis of the infection are probably not correctly followed in a significant number of cases. The same results were previously found in the case of the treatment recommendations [18].

It is known that chronic *H. pylori* infection leads to clinical complications such as peptic ulcers or gastric cancer [4]. In order to reduce these harmful effects, several treatment strategies have been developed. The most widespread, cost-effective, and recommended at a global level test is the so-called “test-and-treat” [6,19,20,21], where those patients with dyspeptic symptoms, in the absence of alarm symptoms and meeting the age range (generally <50 years) should undergo a non-invasive test in order to detect *H. pylori*. However, in patients with alarm symptoms or in those over 50 years old, an upper gastrointestinal endoscopy is recommended in order to exclude potential organic diseases [13,14].

Our study showed that the “test-and-treat” strategy was not followed in all cases, as an invasive test was performed in over half of the patients under the age of 50. Although a proportion of these patients could have reported alarm symptoms, it has been described that these symptoms are usually present in under 5% [22], meaning that in a high percentage of cases, invasive tests might be performed unnecessarily, with the cost and risk this might entail.

Furthermore, in our cohort, an invasive test was not performed in a non-negligible percentage of patients older than 50; thus, upper gastrointestinal endoscopy was not performed to exclude gastric pathology, mainly neoplastic.

The current recommendations state that UBT is the best test to establish the initial diagnosis by non-invasive testing [6]. When UBT is not available, MSAT is also acceptable and presents sensitivity and specificity rates similar to those of UBT [7,8]. Although some serology tests have acceptable rates of sensitivity and specificity, their accuracy may be different depending on the geographic locations and according to the structure of the circulating strains. In this sense, serological tests are accepted only when local validation is achieved; otherwise, their use is not recommended. Other tests such as rapid serology or saliva tests are not recommended either for the initial diagnosis or for the confirmation of the eradication [6,9].

Our study showed that in most of the cases in which a non-invasive test was indicated, a UBT was performed, but a surprisingly low number of cases were diagnosed by MSAT, despite having good diagnostic accuracy. With regards to serology, our study showed it was performed in approximately the same number of cases as MSAT, and local validation is not frequently performed in most centres. This might mean that serology was used without previous proper validation. Our analyses also showed that in a great number of cases, both invasive and non-invasive tests were indicated to establish the initial diagnosis of the infection. This strategy is not generally recommended due to its high cost, the increased risks of complications (i.e., perforation, sedation-related complications, etc.), and the discomfort caused to the patient when performing an unnecessary invasive test [6,19,21].

Culture-guided tailored treatment remains controversial, as there is scarce evidence supporting this strategy [12]. This approach arises from the fact that antibiotic *H. pylori* resistance has increased to alarming levels and local surveillance networks should select appropriate, adapted eradication regimens in each region [23]. Some studies have recommended that the treatment should be selected according to systematic antimicrobial susceptibility testing [24,25]; but the generalised use of susceptibility-guided therapy for *H. pylori* treatment in routine clinical practice, either as first-line or as rescue treatment, is not recommended due to low cost-effectiveness and questionable clinical benefit as compared to empirical highly effective quadruple therapies [10,11,26]. The results obtained in our study showed that in general, culture was indicated in a minority of patients and that most of the cultures were performed in rescue treatment patients, mainly in third-line and subsequent-line treatments, which is consistent with the current recommendations [6]. It must be noted that molecular methods such as real-time PCR were rarely performed during this period, because there was a lack of information on the availability of commercial tests and thermocyclers to perform these tests.

Finally, to confirm the eradication of *H. pylori*, non-invasive testing is recommended in the majority of cases, especially UBT, although MSAT can be a valid alternative when the former is not available [6]. In some exceptional cases, endoscopy is required for other reasons such as checking the healing of gastric ulcer or MALT lymphoma. In these cases, histology is recommended for the evaluation of the eradication, and not RUT as the unique confirmation test [6,19,21].

Our study showed that the methods used to evaluate the eradication in Europe were mainly non-invasive, but a significant number of patients had likewise undergone endoscopy for this purpose. Moreover, RUT was the only test performed in a non-negligible number of patients in this group. This is not consistent with the current guidelines which do not recommend using RUT to assess the *H. pylori* status after an eradication treatment.

With regards to the evolution in the use of tests throughout the years, no clear trend could be observed globally or in the countries with more than 1000 patients included. The COVID pandemic may have influenced the number of UBT and MSAT in 2020. However, this is not clearly shown in our data, and solid conclusions cannot therefore be drawn. More specific time-trend studies should be performed in this field.

Finally, our conclusions are based on assuming all investigators followed the recommendations stablished by the Maastricht VI Consensus Report; however, the countries’ reported differences might be certainly due to specific healthcare or socioeconomic burdens of each setting or variability in health insurance accessibilities. All these might result in high-testing vs. low-testing practices (ultimately following each particular clinical decision); in the use of other *H. pylori*-testing including endoscopy (resulting from the different facilities in each hospital even within the same country); in educational differences (for instance on the knowledge about the accuracy of the different tests; or the misuse of serology as a confirmation test).

Our study has some limitations. As the patients’ alarm symptoms were not registered, we cannot ensure that those undergoing gastroscopy were properly selected. However, previous investigations support that it can be assumed that the presence of “red flags” is infrequent and, therefore, a maximum prevalence of 5% can be estimated [13]. Consequently, the “test-and-treat” strategy is undoubtedly underused [22]. A further limitation is that the age threshold used for the analyses might be debatable. Currently, there is a lack of convincing data supporting a specific cut-off age for endoscopy; therefore, the decision remains somewhat arbitrary. However, setting the age threshold at 55 years seems reasonable in most European countries given the incidence of gastric cancer in this population [13]. For the purpose of this study, the threshold was established at 50 years rather than 55, so it may be assumed that an invasive test was not indicated because of the patients’ age in the absence of red flag symptoms. Another limitation inherent to the design of the registry is that over 70% of the data analysed come from only four countries. However, the results of these countries were analysed separately, in order to focus on the results’ interpretation of their real clinical practice, information that has been also reported in the Supplementary Material. Moreover, we believe that the sample size is sufficient in order to represent the current clinical practice in many European gastroenterologists. We believe the Hp-EuReg data set used is representative of the sample since all variables collected a priori in the patient population mirror the target population; which allows us to draw reliable conclusions as published in the different studies performed to date. Additionally, the sample source, although heterogeneous which can be also seen as a drawback of representativeness, is a very large dataset minimizing any possible bias in the population.

Despite these limitations, our study, the first to analyse the use of diagnostic tests for *H. pylori* infection in Europe, and the largest series including over 35,000 patients from 20 different European countries, provided valuable information that can be used to audit and improve our clinical practice.

In summary, a great heterogeneity between European countries was observed, both in the choice of the pre-treatment diagnostic tests and in the evaluation of the post-treatment eradication of *H. pylori* infection. These results suggest that adherence to the main recommendations on *H. pylori* diagnosis might be able to be improved. The reasons for this apparent lack of adherence to the current clinical practice guidelines should be further clarified and addressed.

## Figures and Tables

**Table 1:** Demographic characteristics of included patients.

		Overall n (%) N = 34,920	Treatment-naïve n (%) N = 27,776	Rescue Treatments n (%) N = 7144
Age; years (Mean (±SD))		51.0 (13.7)	50.3 (15.1)	50.4 (14.2)
Age	<50 years ≥50 years	16,467 (47.2) 18,400 (52.8)	13,179 (47.5) 14,554 (52.5)	3288 (46.1) 3846 (53.9)
Gender	Female Male	21,350 (61.2) 13,545 (38.8)	16,677 (60.1) 11,079 (39.9)	4673 (65.5) 2466 (34.5)
Ethnicity	Caucasian	31,058 (89.1)	24,611 (88.8)	6447 (90.5)
	Black	272 (0.8)	193 (0.7)	79 (1.1)
	Asian	420 (1.2)	330 (1.2)	90 (1.3)
	Other	2299 (6.6)	1973 (7.1)	326 (4.6)
	Not available	796 (2.3)	615 (2.2)	181 (2.5)

**Table 2:** Test used to establish the initial diagnosis of *H. pylori* infection in treatment-*naïve* patients.

	n (% *)
Non-invasive test	11,369 (40.9)
^13^C-urea breath test	7472 (26.9)
^14^C-urea breath test	115 (0.4)
Serology	1824 (6.6)
Monoclonal stool antigen test	1915 (6.9)
Polyclonal stool antigen test	282 (1)
Invasive test	19,801 (71.3)
Histology	11,885 (42.8)
Rapid urease test	10,636 (38.3)
Culture	2927 (10.5)
Biochemical methods (PCR, FISH)	265 (1)

* Out of 27,776 treatment-*naïve* patients (please note that the number of tests is not the same as the number of patients, because in some of the cases, more than one test was conducted).

**Table 3:** Invasive tests used for the initial *H. pylori* diagnosis in treatment-*naïve* patients according to patient’s age.

	Patients with Invasive Diagnostic Test, n/N (%)	Patients <50 yo with Invasive Diagnostic Test, n/N <50 yo (%)	Patients ≥50 yo with Invasive Diagnostic Test, n/N ≥50 yo (%)	*p*-Value
Azerbaijan	565/570 (99.1)	382/386 (99.0)	183/184 (99.5)	1.000
Croatia	277/338 (82.0)	70/99 (70.7)	207/239 (86.6)	0.001 *
France	101/107 (94.4)	46/49 (93.9)	55/58 (94.8)	1.000
Germany	101/132 (76.5)	40/55 (72.7)	61/77 (79.2)	0.386
Greece	497/541 (91.9)	184/211 (87.2)	313/330 (94.8)	0.002 *
Hungary	194/233 (83.3)	77/95 (81.1)	117/138 (84.8)	0.454
Ireland	221/313 (70.6)	90/164 (54.9)	131/149 (87.9)	<0.001 *
Israel	59/103 (57.3)	21/52 (40.4)	38/51 (74.5)	<0.001 *
Italy	2213/2629 (84.2)	904/1117 (80.9)	1300/1485 (87.5)	<0.001 *
Latvia	426/528 (80.7)	250/326 (76.7)	176/202 (87.1)	0.003 *
Lithuania	397/512 (77.5)	149/203 (73.4)	248/309 (80.3)	0.069
Norway	598/740, (80.8)	215/261 (82.4)	383/479 (80.0)	0.425
Portugal	337/347 (97.1)	103/107 (96.3)	233/239 (97.5)	0.506
Russia	3520/5245 (67.1)	1871/2879 (65.0)	1648/2364 (69.7)	<0.001 *
Serbia	67/92 (72.8)	16/31 (51.6)	51/61 (83.6)	0.001 *
Slovenia	2304/2411 (95.6)	952/983 (96.8)	1352/1428 (94.7)	0.011 *
Spain	7482/12,331 (60.7)	3027/5876 (51.5)	4447/6442 (69.0)	<0.001 *
Turkey	247/264 (93.6)	137/150 (91.3)	110/114 (96.5)	0.091
United Kingdom	98/195 (50.3)	18/62 (29.0)	80/133 (60.2)	<0.001 *
Ukraine	97/145 (66.9)	51/73 (69.9)	46/72 (63.9)	0.445
TOTAL	19,801/27,776 (71.3)	8603/13,179 (65.3)	11,179/14,554 (76.8)	<0.001 *

* *p*-value < 0.05. n = number of patients in which an invasive test was performed; N = total number of patients by country.

**Table 4:** Test performed for initial diagnosis of *H. pylori* infection according to patient’s characteristics.

		Non-Invasive Diagnostic Test	Invasive Diagnostic Test	*p*-Value
Age, mean (25–75th percentiles) (continuous) *		46 [(35–58)	53 (41–63)	<0.001 **
Age, n (%) (categorical)	<50 years	4576 (39.7)	6955 (60.3)	<0.001 **
	≥50 years	3375 (26.3)	9435 (73.7)	
Gender, n (%)	Female	5028 (34.3)	9627 (65.7)	<0.001 **
	Male	2943 (30.3)	6768 (69.7)	
Ethnic background, n (%)	Caucasian	6776 (30.9)	15,137 (69.1)	0.509
	Black	45 (31.7)	97 (68.3)	
	Asian	72 (34.6)	136 (65.4)	
	Azerbaijan	5 (0.9)	564 (99.1)	
Country, n (%)	Croatia	61 (18.3)	273 (81.7)	<0.001 **
	France	6 (5.9)	96 (94.1)	
	Germany	31 (25.6)	90 (74.4%)	
	Greece	44 (8.5)	474 (91.5)	
	Hungary	39 (27.5)	103 (72.5)	
	Ireland	92 (29.7)	218 (70.3)	
	Israel	44 (44.4)	55 (55.6)	
	Italy	416 (49.8)	420 (50.2)	
	Latvia	102 (19.7)	416 (80.3)	
	Lithuania	115 (22.9)	387 (77.1)	
	Norway	142 (21.8)	510 (78.2)	
	Portugal	10 (2.9)	336 (97.1)	
	Russia	1725 (40)	2585 (60)	
	Serbia	25 (29.1)	61 (70.9)	
	Slovenia	107 (4.7)	2170 (95.3)	
	Spain	4849 (40.2)	7218 (59.8)	
	Turkey	17 (6.5)	245 (93.5)	
	United Kingdom	97 (51.1)	93 (48.9)	
	Ukraine	48 (34)	93 (66)	

* Not normal distribution. Expressed by median and 25–75th percentiles. ** *p*-value < 0.05.

**Table 5:** Control tests used post-treatment to confirm *H. pylori* eradication.

	Overall, n (%) N = 34,920	First-Line, n (%) N = 27,776	Rescue Treatments, n (%) N = 7144
Non-invasive test	32,540 (93.2)	25,772 (92.8)	6768 (94.7)
^13^C-urea breath test	27,320 (78.2)	21,297 (76.7)	6023 (84.3)
^14^C-urea breath test	389 (1.1)	322 (1.2)	67 (0.9)
Serology	388 (1.1)	302 (1.1)	86 (1.2)
Monoclonal stool antigen test	3673 (10.5)	3117 (11.2)	556 (7.8)
Polyclonal stool antigen test	1259 (3.6)	1172 (4.2)	87 (1.2)
Invasive test	2983 (8.5)	2458 (8.8)	525 (7.3)
Histology	1887 (5.4)	1533 (5.5)	354 (5.0)
Rapid Urease Test	1223 (3.5)	1040 (3.7)	183 (2.6)

Rescue treatments: second, third, and further lines of treatment.

## Data Availability

All data relevant to the study are included in the article or uploaded as Supplementary Information. The data supporting the conclusions of this study are not publicly available, as their content may compromise the privacy of research participants. However, previous published data on the Hp-EuReg study, or de-identified raw data referring to current study, as well as further information on the methods used to explore the data could be shared, with no particular time constraint. Individual participant data will not be shared.

## Supplementary Materials

The following supporting information can be downloaded at: https://www.mdpi.com/article/10.3390/jcm12134363/s1, Supplementary File S1. Hp-EuReg investigators; Table S1: Patients included by European country; Table S2: Pre-treatment concomitant invasive tests (histology and rapid urease test) performed for *H. pylori* initial diagnosis. Table S3: Distribution by country of type of test used for initial diagnosis of H. pylori in treatment-*naïve* patients; Table S4: Evolution of tests used for initial diagnosis in Europe by year; Table S5: Evolution of tests used for initial diagnosis in those European countries with >1000 patients by year; Table S6: Post-treatment concomitant invasive (histology and rapid urease test) control tests to confirm *H. pylori* eradication; Table S7: Type of test used by country for confirmation of eradication of *H. Pylori*; Table S8: Evolution of control tests used in Europe by year; Table S9: Evolution of control tests used in the most representative countries (defined by >1000 patients included) by year; Table S10: Patients with culture according to line of treatment and country.
