# Supplementary material for: Helicobacter pylori Diagnostic Tests Used in Europe: Results of over 34,000 Patients from the European Registry on Helicobacter pylori Management
Source: J Clin Med. 2023 Jun 28;12(13):4363. doi: 10.3390/jcm12134363 (PMC10342434; doi:10.3390/jcm12134363)
Supplement: Supplementary file 1 [file jcm-12-04363-s001.zip › jcm-2455148-supplementary.pdf]

SUPPLEMENTARY MATERIAL:

Supplementary File S1. Hp-EuReg investigators.

- Manuel Pabon Carrasco, Hospital Valme, Sevilla, Spain, acquired data, critically reviewed the manuscript draft, and approved the submitted manuscript.
- Luis Bujanda, Hospital de Donosti and Centro de Investigación Biomédica en Red de Enfermedades Hepáticas y Digestivas (CIBERehd), San Sebastián, Spain, acquired data, critically reviewed the manuscript draft, and approved the submitted manuscript.
- Yanire Brenes, Hospital Universitario de La Princesa, Instituto de Investigación Sanitaria Princesa (IIS-Princesa), Universidad Autónoma de Madrid (UAM) and Centro de Investigación Biomédica en Red de Enfermedades Hepáticas y Digestivas (CIBERehd), Madrid, Spain, acquired data, critically reviewed the manuscript draft, and approved the submitted manuscript.
- Ana Isabel Garre, Hospital Universitario de La Princesa, Instituto de Investigación Sanitaria Princesa (IIS-Princesa), Universidad Autónoma de Madrid (UAM) and Centro de Investigación Biomédica en Red de Enfermedades Hepáticas y Digestivas (CIBERehd), Madrid, Spain, acquired data, critically reviewed the manuscript draft, and approved the submitted manuscript.
- Jennifer Fernandez Pacheco, Hospital Universitario de La Princesa, Instituto de Investigación Sanitaria Princesa (IIS-Princesa), Universidad Autónoma de Madrid (UAM) and Centro de Investigación Biomédica en Red de Enfermedades Hepáticas y Digestivas (CIBERehd), Madrid, Spain, acquired data, critically reviewed the manuscript draft, and approved the submitted manuscript.

- Almudena Durán, Hospital Universitario de La Princesa, Instituto de Investigación Sanitaria Princesa (IIS-Princesa), Universidad Autónoma de Madrid (UAM) and Centro de Investigación Biomédica en Red de Enfermedades Hepáticas y Digestivas (CIBERehd), Madrid, Spain, acquired data, critically reviewed the manuscript draft, and approved the submitted manuscript.
- Maria Caldas, Hospital Universitario de La Princesa, Instituto de Investigación Sanitaria Princesa (IIS-Princesa), Universidad Autónoma de Madrid (UAM) and Centro de Investigación Biomédica en Red de Enfermedades Hepáticas y Digestivas (CIBERehd), Madrid, Spain, acquired data, critically reviewed the manuscript draft, and approved the submitted manuscript.
- Enrique Alfaro Almajano, Hospital Clínico Lozano Blesa, Zaragoza, Spain, acquired data, critically reviewed the manuscript draft, and approved the submitted manuscript.
- Angel Lanas,Hospital Clínico Lozano Blesa, Zaragoza, Spain, acquired data, critically reviewed the manuscript draft, and approved the submitted manuscript.
- Natasa Brglez Jurecic, Diagnostic Center Bled, Slovenia, acquired data, critically reviewed the manuscript draft, and approved the submitted manuscript.
- Noelia Alcaide, Hospital Clínico de Valladolid, Spain, acquired data, critically reviewed the manuscript draft, and approved the submitted manuscript.
- Benito Velayos, Hospital Clínico de Valladolid, Spain, acquired data, critically reviewed the manuscript draft, and approved the submitted manuscript.
- Miguel Areia, Hospital de Coimbra, Portugal, acquired data, critically reviewed the manuscript draft, and approved the submitted manuscript.

- Tatiana Ilchishina, SM-clinic, Saint-Petersburg, Russia, acquired data, critically reviewed the manuscript draft, and approved the submitted manuscript.
- Jorge Pérez, Hospital Universitario de Torrejon, Madrid, Spain, acquired data, critically reviewed the manuscript draft, and approved the submitted manuscript.
- Blas Jose Gomez Rodriguez, Hospital Virgen de la Macarena, Sevilla, Spain, acquired data, critically reviewed the manuscript draft, and approved the submitted manuscript.
- Diego Ledro Cano, Hospital Virgen de la Macarena, Sevilla, Spain, acquired data, critically reviewed the manuscript draft, and approved the submitted manuscript.
- Zdenki Kikec, Hospital Slovenj Gradec, Slovenia, acquired data, critically reviewed the manuscript draft, and approved the submitted manuscript.
- Pavel Bogomolov, Universal clinic - Private medical center, Moscow, Russia, acquired data, critically reviewed the manuscript draft, and approved the submitted manuscript.
- Mónica Perona, Hospital Quiron Marbella, Spain, acquired data, critically reviewed the manuscript draft, and approved the submitted manuscript.
- Igor Bakulin, North-western State Medical University St Petersburg, Russia, acquired data, critically reviewed the manuscript draft, and approved the submitted manuscript.
- Marco Romano, Universita degli Studi della Campania Luigi Vanvitelli, Napoli, Italy, acquired data, critically reviewed the manuscript draft, and approved the submitted manuscript.
- Antonietta Gerarda Gravina, Universita degli Studi della Campania Luigi Vanvitelli, Napoli, Italy, acquired data, critically reviewed the manuscript draft, and approved the submitted manuscript.
- Óscar Núñez, Hospital Univeristario Sanitas La Moraleja- Ntra Sra Rosario, Madrid, Spain, acquired data, critically reviewed the manuscript draft, and approved the submitted manuscript.

- Manuel Domínguez-Cajal, Hospital San Jorge, Huesca, Spain, acquired data, critically reviewed the manuscript draft, and approved the submitted manuscript.
- Liudmila Vologzhanina Gastrocntr, Perm, Russia, acquired data, critically reviewed the manuscript draft, and approved the submitted manuscript.
- Sotirios Georgopoulos, Athens Medical, P. Faliron General Hospital, Athens, Greece, acquired data, critically reviewed the manuscript draft, and approved the submitted manuscript.
- Rinaldo Pellicano, Molinette-SGAS Hospital, Turin, Italy, acquired data, critically reviewed the manuscript draft, and approved the submitted manuscript.
- Rustam Abdulkhakov, Republican clinical hospital of Tatarstan Kazan, Russia, acquired data, critically reviewed the manuscript draft, and approved the submitted manuscript.
- Umud Mahmudov, Modern hospital, Baku, Ganca, Quba, Azerbaijan, acquired data, critically reviewed the manuscript draft, and approved the submitted manuscript.
- Pedro Almela Notari, Hospital General de Castellón, Spain, acquired data, critically reviewed the manuscript draft, and approved the submitted manuscript.
- Leticia Gimeno Pitarch, Hospital General de Castellón, Spain, acquired data, critically reviewed the manuscript draft, and approved the submitted manuscript.
- Gema Ladrón Villanueva, Hospital General de Castellón, Spain, acquired data, critically reviewed the manuscript draft, and approved the submitted manuscript.
- Elida Oblitas, Consorci Sanitari de Terrassa, Barcelona, Spain, acquired data, critically reviewed the manuscript draft, and approved the submitted manuscript.

- Vassiliki Ntouli, General Hospital Piraeus, Greece, acquired data, critically reviewed the manuscript draft, and approved the submitted manuscript.
- Judith Gomez Camarero, Hospital de Burgos, Spain, acquired data, critically reviewed the manuscript draft, and approved the submitted manuscript.
- Manuel Jimenez Moreno, Hospital de Burgos, Spain, acquired data, critically reviewed the manuscript draft, and approved the submitted manuscript.
- Jose Maria Botargues Bote, Hospital de Bellvitge, Barcelona, Spain, acquired data, critically reviewed the manuscript draft, and approved the submitted manuscript.
- Nikola Perkovic, University Hospital Centre Split, Croatia, acquired data, critically reviewed the manuscript draft, and approved the submitted manuscript.
- Goran Hauser, University Hospital Centre Split, Croatia, acquired data, critically reviewed the manuscript draft, and approved the submitted manuscript.
- Cem Simsek Hacettepe, University School of Medicine, Ankara, Turkey, acquired data, critically reviewed the manuscript draft, and approved the submitted manuscript.
- Halis Simsek Hacettepe, University School of Medicine, Ankara, Turkey, acquired data, critically reviewed the manuscript draft, and approved the submitted manuscript.
- Francesco Franceschi, Medicina Interna e Gastroenterologia, Fondazione Policlinico Universitario A. Gemelli IRCCS, Università Cattolica del Sacro Cuore, Roma, Italy, acquired data, critically reviewed the manuscript draft, and approved the submitted manuscript.
- Natalia Nikolaevna Dekhnich, Institute of Antimicrobial Chemotherapy Smolensk, Russia, acquired data, critically reviewed the manuscript draft, and approved the submitted manuscript.

- Emin Mammadov, Memorial klinika (Baku, Ganca, Quba), Azerbaijan, acquired data, critically reviewed the manuscript draft, and approved the submitted manuscript.
- Umud Mahmudov, Memorial klinika (Baku, Ganca, Quba), Azerbaijan, acquired data, critically reviewed the manuscript draft, and approved the submitted manuscript.
- Vendel Kristensen, Lovisenberg Hospital, Oslo, Norway, acquired data, critically reviewed the manuscript draft, and approved the submitted manuscript.
- Rosa Rosania, Otto-von-Guericke University Hospital, Germany, acquired data, critically reviewed the manuscript draft, and approved the submitted manuscript.
- Alexander Link, Otto-von-Guericke University Hospital, Germany, acquired data, critically reviewed the manuscript draft, and approved the submitted manuscript.
- Peter Malfertheiner, Otto-von-Guericke University Hospital, Germany, acquired data, critically reviewed the manuscript draft, and approved the submitted manuscript.
- Rafael Ruiz-Zorrilla, Hospital de Sierrallana, Spain, acquired data, critically reviewed the manuscript draft, and approved the submitted manuscript.
- Galina N. Tarasova, Rostov University, Russia, acquired data, critically reviewed the manuscript draft, and approved the submitted manuscript.
- Miguel Fernández Bermejo, Clinica San Francisco, Cáceres, Spain, acquired data, critically reviewed the manuscript draft, and approved the submitted manuscript.
- Marina Fedorovna Osipenko, City Gastroenterological center Novosibirsk, Russia, acquired data, critically reviewed the manuscript draft, and approved the submitted manuscript.

- Marinko Masuric, University Hospital Sveti Duh, Zagreb, Croatia, acquired data, critically reviewed the manuscript draft, and approved the submitted manuscript.
- M. Anatolevna Livzan, Omsk State Medical Academy of the Ministry of Healthcare, Russia, acquired data, critically reviewed the manuscript draft, and approved the submitted manuscript.
- Deidre McNamara, Tallaght Hospital, Dublin, Ireland, acquired data, critically reviewed the manuscript draft, and approved the submitted manuscript.
- Sinead Smith, University Hospital Policlinico Consorziale, Italy, acquired data, critically reviewed the manuscript draft, and approved the submitted manuscript.
- Ian Beales, University of east anglia, United Kingdom, acquired data, critically reviewed the manuscript draft, and approved the submitted manuscript.
- Pedro Delgado, Hospital de Mérida, Spain, acquired data, critically reviewed the manuscript draft, and approved the submitted manuscript.
- Alain huerta-Madrigal, Hospital de Galdakao-Usansolo, Spain, acquired data, critically reviewed the manuscript draft, and approved the submitted manuscript.
- Farid Vidadi Guliyev, Prime hospital, Baku, Ganca, Quba, Azerbaijan, acquired data, critically reviewed the manuscript draft, and approved the submitted manuscript.
- Giuseppe Losurdo, University Hospital Policlinico Consorziale, Bari, Italy, acquired data, critically reviewed the manuscript draft, and approved the submitted manuscript.
- Alfredo Di Leo, University Hospital Policlinico Consorziale, Bari, Italy, acquired data, critically reviewed the manuscript draft, and approved the submitted manuscript.

- Eduardo Iyo, Hospital Comarcal de Inca, Mallorca, Spain, acquired data, critically reviewed the manuscript draft, and approved the submitted manuscript.
- Javier Tejedor Tejada, Hospital de Cabuenes, Asturias, Spain, acquired data, critically reviewed the manuscript draft, and approved the submitted manuscript.
- V Tsukanov, Scientific Research Inst of medical problems of North Krasnoyars, Russia, acquired data, critically reviewed the manuscript draft, and approved the submitted manuscript.
- Luis Javier Lamuela Calvo, Hospital Universitario Miguel Servet, Zaragoza, Spain, acquired data, critically reviewed the manuscript draft, and approved the submitted manuscript.
- Natalia Baryshnikova, Fisrt St-Petersburg Pavlov State Medical University, St Petersburg, Russia, acquired data, critically reviewed the manuscript draft, and approved the submitted manuscript.
- Sergey Gennadievich Burkov, Outpatient clinic 3 Moscow, Russia, acquired data, critically reviewed the manuscript draft, and approved the submitted manuscript.
- Natalia Bakanova, Medical Center Mediceya, Izhevsk, Russia, acquired data, critically reviewed the manuscript draft, and approved the submitted manuscript.
- Galina Fadeenko, Ukrainian Academy of Medical Sciences, Ukraine, acquired data, critically reviewed the manuscript draft, and approved the submitted manuscript.
- Serhii Melashchenko, Vinnitsa National Medical University, Ukraine, acquired data, critically reviewed the manuscript draft, and approved the submitted manuscript.
- Bruno Richard Molard, Hospital of Bourdeaux, France, acquired data, critically reviewed the manuscript draft, and approved the submitted manuscript.

- Santiago Frago Larramona, Hospital Santa Barbara, Soria, Spain, acquired data, critically reviewed the manuscript draft, and approved the submitted manuscript.
- Ivan Rankovic, Clinical Center of Serbia, Belgrade, Serbia, acquired data, critically reviewed the manuscript draft, and approved the submitted manuscript.
- Frederic Helluwuaert, Centre Hospitalier Annecy Genvois, Pringy, France, acquired data, critically reviewed the manuscript draft, and approved the submitted manuscript.
- Romanas Zykus, Vilkaviskis regional Hospital, Lithuania, acquired data, critically reviewed the manuscript draft, and approved the submitted manuscript.
- Marta Lozano Lanagrán, Hospital Quirón Salud Málaga, Spain, acquired data, critically reviewed the manuscript draft, and approved the submitted manuscript.
- Theodore Rokkas, Henry Dunant Hospital, Athens, Greece, acquired data, critically reviewed the manuscript draft, and approved the submitted manuscript.
- Juris Pokrotnieks, P.Stradin University Clinical hospital, Latvia, acquired data, critically reviewed the manuscript draft, and approved the submitted manuscript.
- Svetlana Cui, P.Stradin University Clinical hospital, Latvia, acquired data, critically reviewed the manuscript draft, and approved the submitted manuscript.
- Antonio Cuadrado Lavín, Hospital Marques Valdecilla, Santander, Spain, acquired data, critically reviewed the manuscript draft, and approved the submitted manuscript.
- Francisco Javier Zozaya Laregui, Clínica Universitaria Navarra, Pamplona, Spain, acquired data, critically reviewed the manuscript draft, and approved the submitted manuscript.

- Ilze Kikuste, Riga East University Clinical hospital, Riga, Latvia, acquired data, critically reviewed the manuscript draft, and approved the submitted manuscript.
- Arne Lihaug Hoff, Hospital of Alesund, Alesund, Norway, acquired data, critically reviewed the manuscript draft, and approved the submitted manuscript.
- Yolanda Arguedas Lázaro, Hospital Royo Villanova, Zaragoza, Spain, acquired data, critically reviewed the manuscript draft, and approved the submitted manuscript.
- Patricia Sanz Segura, Hospital Royo Villanova, Zaragoza, Spain, acquired data, critically reviewed the manuscript draft, and approved the submitted manuscript.
- Nadiya Byelyayeva Donetsk, National Medical University, Donetsk, Ukraine, acquired data, critically reviewed the manuscript draft, and approved the submitted manuscript.
- Valeriy Kryvy, Crimean State Medical University, Simferopol, Ukraine, acquired data, critically reviewed the manuscript draft, and approved the submitted manuscript.
- Mirjana Kalauz, Clinical Hospital Centre Zagreb, Croatia, acquired data, critically reviewed the manuscript draft, and approved the submitted manuscript.
- Isabel Pérez Martínez, Hospital Central de Asturias, Oviedo, Spain, acquired data, critically reviewed the manuscript draft, and approved the submitted manuscript.
- Christos Liatsos, General Military Hospital, Athens, Greece, acquired data, critically reviewed the manuscript draft, and approved the submitted manuscript.
- Konrads Funka, Riga East University Clinical hospital, Riga, Latvia, acquired data, critically reviewed the manuscript draft, and approved the submitted manuscript.

- Ricardo Araújo Cardoso, Hospital S Teotonico, Centro Hospitalar Tondela, Viseu, Portugal, acquired data, critically reviewed the manuscript draft, and approved the submitted manuscript.
- Michel Selgrad, Klinik und Poliiklinik Regensburg, Germany, acquired data, critically reviewed the manuscript draft, and approved the submitted manuscript.
- Montserrat Planella de Rubinat, Hospital Universitari Arnau de Vilanova, Lleida, Spain, acquired data, critically reviewed the manuscript draft, and approved the submitted manuscript.
- Consuelo Ramírez, Hospital Universitari Arnau de Vilanova, Lleida, Spain, acquired data, critically reviewed the manuscript draft, and approved the submitted manuscript.
- Riccardo Vasapolli, Hospital of the Ludwig Maximilians University of Munich, Germany, acquired data, critically reviewed the manuscript draft, and approved the submitted manuscript.
- Lukas Macke, Hospital of the Ludwig Maximilians University of Munich, Germany, acquired data, critically reviewed the manuscript draft, and approved the submitted manuscript.
- Driffa Moussata, Lyon Sud Hospital, Lyon, France, acquired data, critically reviewed the manuscript draft, and approved the submitted manuscript.
- Nora Dansc, Petz Aladár Teaching Hospital, Győr, Hungary, acquired data, critically reviewed the manuscript draft, and approved the submitted manuscript.
- Alenka Forte, MC Heliks Trbovlje, Slovenia, acquired data, critically reviewed the manuscript draft, and approved the submitted manuscript.
- P. Houcke, Hospital of Lille, France, acquired data, critically reviewed the manuscript draft, and approved the submitted manuscript.
- Francisco José Rancel Medina, Hospital Rio Carrión de Palencia, Palencia, Spain, acquired data, critically reviewed the manuscript draft, and approved the submitted manuscript.

- Patrice Pienkowski, Montauban, France, acquired data, critically reviewed the manuscript draft, and approved the submitted manuscript.
- Rolando Pinho, Centro Hospitalar de Vila Nova de Gaia, Portugal, acquired data, critically reviewed the manuscript draft, and approved the submitted manuscript.
- Antonio Cerezo-Ruiz, Hospital Sierra de Segura, Jaen, Spain, acquired data, critically reviewed the manuscript draft, and approved the submitted manuscript.
- Mikhail Butov, Municipal Clinical Hospital N4 Ryazan, Russia, acquired data, critically reviewed the manuscript draft, and approved the submitted manuscript.
- Oleksiy Gridnyev, L.T.Malaya Therapy National Institute of the National Academy of Medical Sciences, Kharkiv, Ukraine, acquired data, critically reviewed the manuscript draft, and approved the submitted manuscript.
- Jonathan Hirsch, Meir Medical Center, Israel, acquired data, critically reviewed the manuscript draft, and approved the submitted manuscript.
- Sandra Agudo, Hospital Rey Juan Carlos I, Mostoles, Madrid, Spain, acquired data, critically reviewed the manuscript draft, and approved the submitted manuscript.
- Angel Cedeño, Hospital Comarcal Sant Jaume de Calella, Barcelona, Spain, acquired data, critically reviewed the manuscript draft, and approved the submitted manuscript.
- Leyanira Torrealba Medina, Hospital Universitario Dr. Josep Trueta, Girona, Spain, acquired data, critically reviewed the manuscript draft, and approved the submitted manuscript.
- Leonardo Henry Eusebi, Policlinico S. Orsola-Malpighi Bologna, Italy, acquired data, critically reviewed the manuscript draft, and approved the submitted manuscript.
- Natalya Marchenko, North-Western State Medical University n.a. I.I.Mechnicov, Russia, acquired data, critically reviewed the manuscript draft, and approved the submitted manuscript.

- Sergiy Svystun, Zaporizzhya State Medical University, Ukraine, acquired data, critically reviewed the manuscript draft, and approved the submitted manuscript.

Table S1. Patients included by European country.

Country	Overall n (%) n=34,920	*Naïve* n (%) n=27,776	Rescue Treatments n (%) n=7,144
Azerbaijan	570 (1.6)	570 (2.1)	0 (0)
Croatia	380 (1.1)	338 (1.2)	42 (0.6)
France	154 (0.4)	107 (0.4)	47 (0.7)
Germany	171 (0.5)	132 (0.5)	39 (0.5)
Greece	642 (1.8)	541 (1.9)	101 (1.4)
Hungary	292 (0.8)	233 (0.8)	59 (0.8)
Ireland	367 (1.1)	313 (1.1)	54 (0.8)
Israel	211 (0.6)	103 (0.4)	108 (1.5)
Italy	3,731 (10.7)	2,629 (9.5)	1,102 (15.4)
Latvia	600 (1.7)	528 (1.9)	72 (1.0)
Lithuania	620 (1.8)	512 (1.8)	108 (1.5)
Norway	894 (2.6)	740 (2.7)	154 (2.2)
Portugal	462 (1.3)	347 (1.2)	115 (1.6)
Russia	5,856 (16.8)	5,245 (18.9)	611 (8.6)
Serbia	131 (0.4)	92 (0.3)	39 (0.5)
Slovenia	2,765 (7.9)	2,411 (8.7)	354 (5)
Spain	16,276 (46.6)	12,331 (44.4)	3,945 (55.2)
Turkey	290 (0.8)	264 (1.0)	26 (0.4)
Ukraine	177 (0.5)	145 (0.5)	32 (0.4)
United Kingdom	331 (0.9)	195 (0.7)	136 (1.9)

Table S2. Pre-treatment concomitant invasive tests (histology and rapid urease test) performed for *H. pylori* initial diagnosis.

	Patients in whom RUT was not conducted, n (%)	Patients in whom RUT was conducted, n (%)	Total, n (%)
Patients in whom Histology was not conducted	8,692 (31.3)	7,199 (25.9)	15,891 (57.2)
Patients in whom Histology was conducted	8,448 (30.4)	3,437 (12.4)	11,885 (42.8)
Total	17,140 (61.7)	10,636 (38.3)	27,776 (100)

RUT: rapid urease test

Table S3. Distribution by country of type of test used for initial diagnosis of *H. pylori* in treatment-*naïve* patients.

Country	Non-invasive Tests					Invasive Tests			
	^{13}C- Urea Breath Test	^{14}C-Urea Breath Test	Serology	Monoclonal Antigen Stool Test	Polyclonal Antigen Stool Test	Histology	Rapid Urease Test	Culture	Biochemical methods (PCR, FISH)
**Azerbaijan**	0 (0)	0 (0)	1 (16.7)	3 (50)	2 (33.3)	0 (0)	565 (100)	0 (0)	0 (0)
**Croatia**	1 (1.6)	0 (0)	0 (0)	63 (98.4)	0 (0)	162 (57.7)	115 (40.9)	4 (1.4)	0 (0)
**France**	9 (75)	0 (0)	3 (25)	0 (0)	0 (0)	99 (90)	0 (0)	7 (6.4)	4 (3.6)
**Germany**	23 (46)	0 (0)	9 (18)	16 (32)	2 (4)	89 (57.1)	63 (40.4)	4 (2.6)	0 (0)
**Greece**	54 (80.6)	0 (0)	10 (14.9)	1 (1.5)	2 (3)	201 (25.8)	347 (44.6)	228 (29.3)	2 (0.3)
**Hungary**	57 (42.5)	5 (3.7)	72 (53.2)	0 (0)	0 (0)	165 (45.8)	107 (29.7)	2 (0.6)	86 (23.9)
**Ireland**	94 (98.9)	0 (0)	0 (0)	0 (0)	1 (1.1)	175 (41)	216 (50.6)	35 (8.2)	1 (0.2)
**Israel**	42 (84)	2 (4)	1 (2)	5 (10)	0 (0)	49 (77.8)	12 (19)	1 (1.6)	1 (1.6)
**Italy**	2154 (96.6)	4 (0.2)	6 (0.3)	48 (2.2)	18 (0.8)	2,184 (38.3)	1,752 (30.7)	1,728 (30.3)	44 (0.8)
**Latvia**	47 (41.2)	0 (0)	66 (57.9)	0 (0)	0 (0)	133 (30.5)	303 (69.5)	0 (0)	0 (0)

**Lithuania**	0 (0)	1 (0.7)	107 (79.9)	15 (11.2)	10 (7.5)	120 (29.7)	283 (70)	1 (0.2)	0 (0)
**Norway**	17 (7.1)	0 (0)	213 (88.8)	7 (2.9)	2 (0.8)	73 (11.3)	218 (33.6)	357 (55.1)	0 (0)
**Portugal**	2 (16.7)	2 (16.7)	7 (58.5)	0 (0)	0 (0)	337 (99.4)	1 (0.3)	1 (0.3)	0 (0)
**Russia**	1050 (37.4)	9 (0.3)	1,133 (40.3)	444 (15.8)	172 (6.1)	1,665 (42.5)	2,068 (52.8)	62 (1.6)	120 (3.1)
**Serbia**	10 (30.3)	2 (6.1)	1 (3)	18 (54.6)	1 (3)	50 (71.4)	16 (22.9)	0 (0)	4 (5.7)
**Slovenia**	217 (89.7)	5 (2.1)	5 (2.1)	15 (6.2)	0 (0)	698 (26)	1,857 (69.2)	128 (4.8)	0 (0)
**Spain**	3,672 (71.4)	63 (1.2)	95 (1.8)	1,245 (24.2)	70 (1.4)	5,475 (65.9)	2,463 (29.6)	367 (4.4)	3 (0.0)
**United Kingdom**	3 (2.8)	4 (3.8)	67 (63.2)	31 (29.2)	1 (0.9)	53 (47.7)	57 (51.4)	1 (0.9)	0 (0)
**Ukraine**	19 (36.5)	0 (0)	28 (53.8)	4 (7.7)	1 (1.9)	48 (48)	51 (51)	1 (1)	0 (0)
**Turkey**	1 (5.3)	18 (94.7)	0 (0)	0 (0)	0 (0)	109 (43.4)	142 (56.6)	0 (0)	0 (0)

Table S4. Evolution of tests used for initial diagnosis in Europe by year.

Test	Year; n (% in the year)									Overall number of tests; n (%)	
	2013	2014	2015	2016	2017	2018	2019	2020	2021		p
^{13}C Urea Breath Test	855 (26.5)	1,103 (25.7)	1,045 (28.4)	1,014 (23.7)	932 (26.4)	956 (28.4)	942 (31.9)	586 (25.3)	17 (20.2)	7,472 (26.9)	< 0.001*
^{14}C Urea Breath Test	6 (0.2)	27 (0.6)	18 (0.5)	45 (1.1)	7 (0.2)	2 (0.1)	6 (0.2)	4 (0.2)	0 (0)	115 (0.4)	< 0.001*
Serology	192 (5.9)	319 (7.4)	217 (5.9)	318 (7.4)	249 (7)	307 (9.1)	135 (4.6)	84 (3.6)	3 (3.6)	1,824 (6.6)	< 0.001*
Monoclonal stool antigen test	140 (4.3)	209 (4.9)	238 (6.5)	346 (8.1)	301 (8.5)	233 (6.9)	257 (8.7)	184 (8)	7 (8.3)	1,915 (6.9)	< 0.001*
Polyclonal stool antigen test	12 (0.4)	9 (0.2)	10 (0.3)	24 (0.6)	8 (0.2)	27 (0.8)	148 (5)	41(1.8)	3 (3.6)	282 (1)	< 0.001*
Histology	1,364 (42.2)	1,863 (43.3)	1,454 (39.5)	1,985 (46.4)	1,778 (50.3)	1,412 (42)	1,172 (39.6)	801 (34.6)	44 (52.4)	11,885 (42.8)	< 0.001*
Rapid urease test	1,243 (38.5)	1,507 (35.1)	1,396 (37.9)	1,365 (31.9)	1,224 (34.6)	1,395 (41.5)	1,234 (41.7)	1,250 (54)	15 (17.9)	10,636 (38.3)	< 0.001*
Culture	410 (12.7)	553 (12.9)	503 (13.7)	320 (7.5)	349 (9.9)	332 (9.9)	278 (9.4)	179 (7.7)	2 (2.4)	2,927 (10.5)	< 0.001*
Biochemical methods (PCR, FISH)	27 (0.8)	67 (1.6)	65 (1.8)	41 (1)	5 (1.2)	2 (0.1)	2 (0.1)	17 (0.7)	0 80)	265 (1)	< 0.001*

* P-value < 0.05

Table S5. Evolution of tests used for initial diagnosis in those European countries with >1,000 patients by year.

Spain (n=16,276)

Test	Year; n (% in the year)									Overall number of tests; n (%)	p
	2013	2014	2015	2016	2017	2018	2019	2020	2021		
^{13}C Urea Breath Test	528 (34)	649 (30.3)	601 (30.2)	572 (27.6)	452 (25.5)	275 (25.7)	282 (32.4)	298 (37.1)	15 (22.4)	3672 (29.8)	< 0.001*
^{14}C Urea Breath Test	0 (0)	3 (0.1)	10 (0.5)	42 (2)	5 (0.3)	1 (0.1)	0 (0)	2 (0.2)	0 (0)	63 (0.5)	< 0.001*
Serology	17 (1.1)	23 (1.1)	19 (1)	19 (0.9)	11 (0.6)	2 (0.2)	4 (0.5)	0 (0)	0 (0)	95 (0.8)	0.53
Monoclonal stool antigen test	109 (7.1)	190 (8.9)	210 (10.6)	186 (9)	212 (12)	137 (12.8)	115 (13.2)	80 (10)	6 (9)	1,245 (10.1)	< 0.001*
Polyclonal stool antigen test	10 (0.6)	6 (0.3)	6 (0.3)	8 (0.4)	6 (0.3)	9 (0.8)	15 (1.7)	7 (0.9)	3 (4.5)	70 (0.6)	< 0.001*
Histology	657 (42.6)	1,037 (48.5)	765 (38.4)	886 (42.8)	835 (47.1)	488 (45.5)	372 (42.8)	394 (49)	41 (59.7)	5,475 (44.4)	< 0.001*
Rapid urease test	309 (19.9)	366 (17.1)	430 (21.6)	470 (22.7)	356 (20.1)	210 (19.6)	133 (15.3)	183 (22.8)	6 (9)	2,463 (20)	< 0.001*
Culture	12 (0.8)	108 (5)	124 (6.2)	73 (3.5)	21 (1.2)	11 (1)	14 (1.6)	4 (0.5)	0 (0)	367 (3)	< 0.001*
Biochemical methods (PCR, FISH)	0 (0)	0 (0)	0 (0)	1 (0.1)	0 (0)	1 (0.1)	0 (0)	1 (0.1)	0 (0)	3 (0.1)	0.72

* P-value < 0.05

Russia (n=5,856)

Test	Year; n (% in the year)									Overall number of tests; n (%)	p
	2013	2014	2015	2016	2017	2018	2019	2020	2021		
^{13}C Urea Breath Test	20 (11.)	40 (11.4)	79 (19)	114 (13.1)	63 (9.4)	290 (22.2)	334 (31)	105 (31)	1 (25)	1050 (20)	< 0.001*
^{14}C Urea Breath Test	0 (0)	0 (0)	5 (1.4)	3 (0.7)	1 (0.1)	0 (0)	0 (0)	0 (0)	0 (0)	9 (0.2)	< 0.001*
Serology	51 (25.8)	101 (28.8)	97 (23.3)	213 (24.5)	202 (30)	292 (22.3)	116 (10.8)	60 (17.7)	1 (25)	1,133 (21.6)	< 0.001*
Monoclonal stool antigen test	10 (5.1)	4 (1.1)	12 (2.9)	132 (15.2)	74 (11)	58 (4.4)	89 (8.2)	65 (19.2)	0 (0)	444 (8.5)	< 0.001*
Polyclonal stool antigen test	1 (0.5)	2 (0.6)	2 (0.5)	4 (0.5)	0 (0)	9 (0.7)	126 (11.7)	28 (8.3)	0 (0)	172 (3.3)	< 0.001*
Histology	62 (31.3)	51 (14.5)	129 (31)	440 (50.6)	304 (45.2)	359 (27.4)	222 (20.6)	97 (28.3)	0 (0)	1,665 (31.7)	< 0.001*
Rapid urease test	77 (38.9)	184 (52.4)	137 (32.9)	231 (26.6)	182 (27)	562 (42.9)	575 (53.3)	116 (34.2)	2 (50)	2,068 (39.4)	< 0.001*
Culture	18 (9.1)	33 (9.4)	2 (0.5)	0 (0)	2 (0.3)	7 (0.5)	0 (0)	0 (0)	0 (0)	62 (1.2)	< 0.001*
Biochemical methods (PCR, FISH)	16 (8.1)	34 (9.7)	50 (12)	16 (1.8)	0 (0)	4 (0.3)	0 (0)	0 (0)	0 (0)	120	120 (2.3)

* P-value < 0.05

Italy (n=3,731)

Test	Year; n (% in the year)									Overall number of tests; n (%)	
	2013	2014	2015	2016	2017	2018	2019	2020	2021		p
^{13}C Urea Breath Test	198 (85.3)	246 (84.2)	289 (95.1)	249 (72.2)	373 (82.5)	375 (82.6)	270 (75)	155 (84.1)	1 (33.3)	2,137 (81.8)	< 0.001*
^{14}C Urea Breath Test	1 (0.4)	0 (0)	1 (0.3)	1 (0.3)	1 (0.2)	0 (0)	0 (0)	0 (0)	0 (0)	4 (0.2)	0.89
Serology	2 (0.9)	0 (0)	1 (0.3)	0 (0)	0 (0)	2 (0.5)	1 (0.3)	0 (0)	0 (0)	6 (0.2)	0.5
Monoclonal stool antigen test	3 (0.9)	6 (2.1)	2 (0.7)	13 (3.8)	4 (0.9)	12 (2.8)	7 (1.9)	1 (0.5)	0 (0)	48 (1.8)	0.04*
Polyclonal stool antigen test	0 (0)	0 (0)	0 (0)	7 (2)	1 (0.2)	8 (1.9)	1 (0.3)	1 (0.5)	0 (0)	18 (0.7)	0.02*
Histology	179 (77.5)	273 (93.5)	281 (92.4)	263 (76.2)	386 (85.4)	353 (81.7)	274 (76.1)	163 (88.6)	3 (100)	2,184 (83.1)	< 0.001*
Rapid urease test	150 (64.5)	231 (79.1)	272 (89.5)	182 (52.8)	295 (65.3)	293 (67.8)	194 (53.9)	134 (72.8)	1 (33.3)	1,752 (66.6)	< 0.001*
Culture	150 (64.5)	231 (79.1)	269 (88.5)	179 (51.9)	295 (65.3)	283 (65.5)	192 (53.3)	128 (69.9)	1 (33.3)	1,728 (65.7)	< 0.001*
Biochemical methods (PCR, FISH)	0 (0)	2 (0.7)	0 (0)	1 (0.3)	30 (6.6)	0 (0)	2 (0.6)	9 (4.9)	0 (0)	44 (1,7)	< 0.001*

* P-value < 0.05

Slovenia (n=2,765)

Test	Year; n (% in the year)									Overall number of tests; n (%)	p
	2013	2014	2015	2016	2017	2018	2019	2020	2021		
^{13}C Urea Breath Test	66 (20.1)	21 (4.7)	29 (8)	8 (2.4)	26 (9.5)	15 (5.6)	42 (16.3)	10 (7.6)	0 (0)	219 (9)	< 0.001*
^{14}C Urea Breath Test	0 (0)	3 (0.7)	1 (0.3)	0 (0)	0 (0)	0 (0)	1 (0.4)	0 (0)	0 (0)	5 (0.2)	0.55
Serology	0 (0)	2 (0.4)	1 (0.3)	0 (0)	0 (0)	1 (0.4)	1 (0.4)	0 (0)	0 (0)	5 (0.2)	0.89
Monoclonal stool antigen test	0 (0)	0 (0)	0 (0)	0 (0)	0 (0)	5 (1.9)	9 (3.5)	1 (0.8)	0 (0)	15 (0.6)	< 0.001*
Polyclonal stool antigen test	0 (0)	0 (0)	0 (0)	0 (0)	0 (0)	0 (0)	0 (0)	0 (0)	0 (0)	0 (0)	< 0.001*
Histology	178 (54.1)	185 (41.2)	79 (21.7)	87 (26.1)	21 (7.7)	41 (15.2)	78 (30.2)	29 (22)	0 (0)	698 (29)	< 0.001*
Rapid urease test	240 (72.9)	317 (70.6)	274 (75.3)	239 (71.8)	228 (93.2)	225 (83.3)	204 (79.1)	128 (97)	2 (100)	1,857 (77)	< 0.001*
Culture	45 (13.7)	11 (2.4)	1 (0.3)	0 (0)	0 (0)	7 (2.6)	37 (14.3)	27 (20.5)	0 (0)	128 (5.3)	< 0.001*
Biochemical methods (PCR, FISH)	0 (0)	0 (0)	0 (0)	0 (0)	0 (0)	0 (0)	0 (0)	0 (0)	0 (0)	0 (0)	< 0.001*

* P-value < 0.05

Table S6. Post-treatment concomitant invasive (histology and rapid urease test) control tests to confirm *H. pylori* eradication.

	Patients in whom RUT was not conducted, n (%)	Patients in whom RUT was conducted, n (%)	Total, n (%)
Patients in whom Histology was not conducted	25,378 (91.4)	865 (3.1)	26,243 (94.5)
Patients in whom Histology was conducted	1,358 (4.9)	175 (0.6)	1,533 (5.5)
Total	26,736 (96.3)	1,040 (3.7)	27,776 (100)

RUT: rapid urease test

Table S7. Type of test used by country for confirmation of eradication of *H. Pylori*.

Country	Non-invasive tests					Invasive tests	
	^{13}C Urea Breath Test	^{14}C Urea Breath Test	Serology	Monoclonal antigen stool test	Polyclonal antigen stool test	Histology	Rapid Urease Test
**Azerbaijan**	0 (0)	0 (0)	0 (0)	34 (6.3)	506 (93.7)	0 (0)	31 (100)
**Croatia**	2 (0.5)	3 (0.8)	0 (0)	337 (92.3)	23 (6.3)	12 (75)	4 (25)
**France**	142 (100)	0 (0)	0 (0)	0 (0)	0 (0)	12 (92.3)	1 (7.7)
**Germany**	153 (90)	0 (0)	1 (0.6)	14 (8.2)	2 (1.2)	7 (77.8)	2 (22.2)
**Greece**	575 (98.5)	1 (0.2)	2 (0.3)	2 (0.3)	4 (0.7)	72 (61)	46 (39)
**Hungary**	289 (100)	0 (0)	0 (0)	0 (0)	0 (0)	4 (66.7)	2 (33.3)
**Ireland**	341 (99.4)	1 (0.3)	0 (0)	1 (0.3)	0 (0)	23 (52.3)	21 (47.7)
**Israel**	176 (91.2)	1 (0.5)	0 (0)	15 (7.8)	1 (0.5)	7 (43.8)	9 (56.3)
**Italy**	3551 (95.6)	4 (0.1)	0 (0)	111 (3)	47 (1.3)	66 (68)	31 (32)
**Latvia**	572 (99.8)	0 (0)	0 (0)	0 (0)	1 (0.2)	20 (46.5)	23 (53.5)
**Lithuania**	3 (1.3)	9 (3.9)	76 (33.2)	101 (44.1)	40 (17.5)	212 (48.4)	226 (51.6)

**Norway**	730 (83.3)	0 (0)	10 (1.1)	125 (14.3)	11 (1.3)	23 (65.7)	12 (34.3)
**Portugal**	407 (94.7)	22 (5.1)	0 (0)	1 (0.2)	0 (0)	33 (97.1)	1 (2.9)
**Russia**	2,708 (51.6)	9 (0.2)	284 (5.4)	1,695 (32.3)	554 (10.6)	898 (66.3)	456 (33.7)
**Serbia**	32 (25.8)	7 (5.6)	3 (2.4)	82 (66.1)	0 (0)	6 (60)	4 (40)
**Slovenia**	2,637 (99.2)	6 (0.2)	0 (0)	12 (0.5)	4 (0.2)	62 (54.4)	52 (45.6)
**Spain**	14,783 (92.5)	235 (1.5)	5 (0.0)	902 (5.6)	58 (0.4)	328 (68.5)	151 (31.5)
**United Kingdom**	171 (55.2)	2 (0.6)	3 (1)	129 (41.6)	5 (1.6)	22 (64.7)	12 (35.3)
**Ukraine**	48 (29.6)	0 (0)	4 (2.5)	108 (66.7)	2 (1.2)	10 (62.5)	6 (37.5)
**Turkey**	0 (0)	89 (94.7)	0 (0)	4 (4.3)	1 (1.1)	70 34.5)	133 (65.5)

Table S8. Evolution of control tests used in Europe by year.

Test	Year; n (% in the year)									Overall number of tests; n (%)	p
	2013	2014	2015	2016	2017	2018	2019	2020	2021		
^{13}C Urea Breath Test	3,317 (82.2)	4,822 (85.8)	4,075 (84.3)	4,096 (74.2)	3,378 (77)	3,062 (74.3)	2,888 (79.5)	1,595 (57.5)	73 (67.3)	27,320 (78.2)	< 0.001*
^{14}C Urea Breath Test	50 (1.2)	82 (1.5)	43 (0.9)	119 (2.2)	59 (1.4)	14 (0.3)	19 (0.5)	3 (0.1)	0 (0)	389 (1.1)	< 0.001*
Serology	40 (1)	23 (0.4)	35 (0.7)	100 (1.8)	56 (1.3)	109 (2.7)	10 (0.3)	15 (0.5)	0 (0)	388 (1.1)	< 0.001*
Monoclonal stool antigen test	309 (7.7)	383 (6.8)	309 (6.4)	650 (11.8)	436 (10)	549 (13.4)	512 (14.2)	502 (18.3)	23 (22.8)	3,673 (10.5)	< 0.001*
Polyclonal stool antigen test	22 (0.5)	38 (0.7)	67 (1.4)	186 (3.4)	110 (2.5)	105 (2.6)	145 (4)	577 (21)	8 (6.9)	1,259 (3.6)	< 0.001*
Histology	224 (5.6)	175 (3.1)	213 (4.4)	512 (9.3)	334 (7.6)	187 (4.6)	151 (4.2)	86 (3.1)	5 (4)	1,887 (5.4)	< 0.001*
Rapid urease test	168 (4.2)	176 (3.1)	186 (3.9)	180 (3.3)	133 (3)	173 (4.2)	102 (2.8)	104 (3.7)	1 (1)	1,223 (3.5)	< 0.001*
Culture	27 (0.7)	25 (0.4)	12 (0.2)	16 (0.3)	14 (0.3)	31 (0.8)	5 (0.1)	7 (0.3)	0 (0)	137 (0.4)	0.03*
Biochemical methods (PCR, FISH)	0 (0)	2 (0.01)	1 (0.01)	9 (0.2)	9 (0.2)	68 (1.7)	8 (0.2)	4 (0.1)	0 (0)	101 (0.3)	< 0.001*

* P-value < 0.05

Table S9. Evolution of control tests used in the most representative countries (defined by >1,000 patients included) by year.

Spain (n=16,276)

Test	Year; n (% in the year)									Overall number of tests; n (%)	
	2013	2014	2015	2016	2017	2018	2019	2020	2021		p
^{13}C Urea Breath Test	1,919 (95.2)	2,662 (91.9)	2,487 (91.1)	2,488 (89.9)	2,007 (89.2)	1.229 (91.3)	1,063 (91.9)	867 (84.1)	60 (74.7)	14,782 (90.8)	< 0.001*
^{14}C Urea Breath Test	6 (0.3)	13 (0.4)	38 (1.4)	117 (4.2)	49 (2.2)	10 (0.7)	1 (0.1)	0 (0)	0 (0)	235 (1.4)	< 0.001*
Serology	0 (0)	0 (0)	1 (0.01)	1 (0.01)	1 (0.01)	1 (0.1)	0 (0)	1 (0.1)	0 (0)	5 (0.01)	1
Monoclonal stool antigen test	54 (2.7)	151 (5.2)	169 (6.2)	115 (4.2)	125 (5.6)	81 (6)	58 (5)	130 (12.6)	19 (24.1)	902 (5.5)	< 0.001*
Polyclonal stool antigen test	2 (0.1)	14 (0.5)	1 (0.01)	14 (0.5)	9 (0.4)	8 (0.6)	2 (0.2)	8 (0.8)	0 (0)	58 (0.4)	0.07
Histology	36 (1.7)	59 (2)	34 (1.2)	50 (1.8)	63 (2.8)	28 (2.1)	32 (2.8)	23 (2.2)	3 (3.8)	328 (2)	0.002*
Rapid urease test	19 (0.9)	30 (1)	28 (1)	22 (0.8)	23 (1)	14 (1)	9 (0.8)	6 (0.6)	0 (0)	151 (0.9)	1
Culture	14 (0.5)	11 (0.3)	3 (0.1)	7 (0.2)	8 (0.2)	29 (0.8)	3 (0.1)	3 (0.2)	0 (0)	78 (0.3)	1

* P-value < 0.05

Russia (n=5,856)

Test	Year; n (% in the year)									Overall number of tests; n (%)	
	2013	2014	2015	2016	2017	2018	2019	2020	2021		p
^{13}C Urea Breath Test	49 (22.6)	144 (38.1)	190 (40.3)	235 (22.4)	211 (26.9)	681 (47.7)	904 (78.3)	228 (61.6)	5 (60)	2,648 (45.2)	< 0.001*
^{14}C Urea Breath Test	4 (1.9)	4 (1.1)	0 (0)	0 (0)	0 (0)	1 (0.1)	0 (0)	0 (0)	0 (0)	9 (0.2)	< 0.001*
Serology	31 (14.6)	13 (3.4)	21 (4.5)	84 (8)	30 (3.8)	100 (7)	4 (0.3)	1 (0.3)	0 (0)	284 (4.8)	< 0.001*
Monoclonal stool antigen test	54 (25.5)	132 (34.9)	103 (21.9)	365 (34.8)	264 (33.7)	369 (25.9)	240 (20.8)	167 (45.1)	1 (20)	1,695 (28.9)	< 0.001*
Polyclonal stool antigen test	10 (4.7)	15 (4)	59 (12.5)	162 (15.5)	98 (12.5)	84 (5.9)	93 (8.1)	32 (8.6)	1 (20)	554 (9.5)	< 0.001*
Histology	48 (22.6)	26 (6.9)	85 (18)	391 (37.3)	213 (27.2)	95 (6.7)	33 (2.9)	6 (1.6)	0 (0)	898 (15.3)	< 0.001*
Rapid urease test	37 (17)	63 (16.7)	47 (10)	76 (7.3)	49 (6.3)	111 (7.8)	50 (4.3)	23 (5.9)	0 (0)	459 (7.8)	< 0.001*
Culture	4 (1.9)	4 (1.1)	1 (0.2)	0 (0)	1 (0.1)	9 (0.6)	0 (0)	0 (0)	0 (0)	19 (0.3)	0.001*

* P-value < 0.05

Italy (n=3,731)

Test	Year; n (% in the year)									Overall number of tests; n (%)	p
	2013	2014	2015	2016	2017	2018	2019	2020	2021		
^{13}C Urea Breath Test	328 (96)	465 (95.7)	439 (98.4)	422 (95.3)	538 (98.5)	649 (97.6)	505 (91.7)	202 (82.1)	3 (60)	3,551 (95.2)	< 0.001*
^{14}C Urea Breath Test	1 (0.3)	0 (0)	0 (0)	1 (0.2)	0 (0)	0 (0)	2 (0.4)	0 (0)	0 (0)	4 (0.1)	0.55
Serology	0 (0)	0 (0)	0 (0)	0 (0)	0 (0)	0 (0)	0 (0)	0 (0)	0 (0)	0 (0)	0.55
Monoclonal stool antigen test	12 (3.5)	19 (3.9)	7 (1.6)	18 (4.1)	6 (1.1)	12 (1.8)	14 (2.5)	23 (9.3)	0 (0)	111 (3)	< 0.001*
Polyclonal stool antigen test	2 (0.5)	0 (0)	3 (0.7)	5 (1.1)	0 (0)	6 (0.9)	14 (2.5)	15 (6.1)	2 (40)	47 (1.3)	< 0.001*
Histology	7 (2.2)	5 (1)	3 (0.7)	4 (0.9)	5 (0.9)	19 (2.9)	17 (3.1)	6 (2.4)	0 (0)	66 (1.8)	0.015 *
Rapid urease test	2 (0.6)	1 (0.2)	4 (0.9)	2 (0.5)	3 (0.5)	15 (2.3)	3 (0.5)	1 (0.4)	0 (0)	31 (0.8)	0.01*
Culture	2 (0.6)	1 (0.2)	0 (0)	3 (0.7)	3 (0.5)	19 (2.9)	2 (0.4)	1 (0.4)	0 (0)	31 (0.8)	< 0.001*

* P-value < 0.05

Slovenia (n=2,765)

Test	Year; n (% in the year)									Overall number of tests; n (%)	
	2013	2014	2015	2016	2017	2018	2019	2020	2021		p
^{13}C Urea Breath Test	337 (90.8)	516 (35.4)	413 (96.3)	377 (98.4)	307 (97.5)	293 (96.4)	250 (91.9)	142 (95.9)	2 (100)	2,637 (95.4)	< 0.001*
^{14}C Urea Breath Test	0 (0)	1 (0.2)	4 (0.9)	1 (0.3)	0 (0)	0 (0)	0 (0)	0 (0)	0 (0)	6 (0.2)	0.15
Serology	0 (0)	0 (0)	0 (0)	0 (0)	0 (0)	0 (0)	0 (0)	0 (0)	0 (0)	0 (0)	0.005*
Monoclonal stool antigen test	2 (0.5)	1 (0.2)	0 (0)	1 (0.3)	2 (0.6)	4 (1.3)	2 (0.7)	0 (0)	0 (0)	12 (0.4)	0.35
Polyclonal stool antigen test	0 (0)	0 (0)	0 (0)	0 (0)	0 (0)	0 (0)	3 (1.1)	1 (0.7)	0 (0)	4 (0.1)	0.005*
Histology	21 (5.7)	115 (2.8)	6 (1.4)	1 (0.3)	3 (1)	2 (0.7)	11 (4)	3 (2)	0 (0)	62 (2.2)	< 0.001*
Rapid urease test	12 (3.2)	9 (1.7)	9 (2.1)	6 (1.6)	3 (1)	5 (1.6)	6 (2.2)	2 (1.4)	0 (0)	52 (1.9)	0.73
Culture	6 (1.6)	4 (0.7)	0 (0)	0 (0)	0 (0)	0 (0)	0 (0)	0 (0)	0 (0)	10 (0.4)	0.003*

* P-value < 0.05

Table S10. Patients with culture according to line of treatment and country.

	Patients with culture, n/N (%)	*Naïve* patients with culture, n/N (%)	Patients with culture after first-line treatment, n/N (%)	Patients with culture after second-line treatments, n/N (%)	Patients with culture in rescue treatments, n/N (%)
**Azerbaijan**	0/570 (0)	0/570 (0)	0/0 (0)	0/0 (0)	0/0 (0)
**Croatia**	16/380 (4.2)	4/338 (1.2)	6/31 (19.3)	6/11 (54.5)	12/42 (28.6)
**France**	23/154 (14.9)	7/107 (6.5)	5/24 (20.8)	11/23 (47.8)	16/47 (34.0)
**Germany**	12/171 (7.0)	4/132 (3.0)	5/33 (15.1)	3/6 (50.0)	8/39 (20.5)
**Greece**	249/642 (38.8)	228/541 (42.1)	13/84 (15.5)	8/17 (47.1)	21/101 (20.8)
**Hungary**	2/292 (0.7)	2/233 (0.9)	0/47 (0)	0/12 (0)	0/59 (0)
**Ireland**	43/367 (11.7)	35/313 (11.2)	5/41 (12.2)	3/13 (23.1)	8/54 (14.8)
**Israel**	65/211 (30.8)	1/103 (1.0)	7/33 (21.2)	57/75 (76.0)	64/108 (59.3)
**Italy**	2,475/3,731 (66.3)	1,728/2,629 (65.7)	427/633 (67.5)	320/469 (68.2)	747/1102 (67.8)
**Latvia**	0/600 (0)	0/528 (0)	0/61 (0)	0/11 (0)	0/72 (0)

**Lithuania**	1/620 (0.2)	1/512 (0.2)	0/96 (0)	0/12 (0)	0/108 (0)
**Norway**	406/894 (45.4)	357/740 (48.3)	32/117 (27.3)	17/37 (46.0)	49/154 (31.8)
**Portugal**	15/462 (3.3)	1/347 (0.3)	1/76 (1.3)	13/39 (33.3)	14/115 (12.2)
**Russia**	65/5,856 (1.1)	62/5,245 (1.2)	2/556 (0.4)	1/55 (1.8)	3/611 (0.5)
**Serbia**	3/131 (2.3)	0/92 (0)	2/29 (6.9)	1/10 (10.0)	3/39 (7.7)
**Slovenia**	178/2,765 (6.4)	128/2,411 (5.3)	34/289 (11.8)	16/65 (24.6)	50/354 (14.1)
**Spain**	422/16,276 (2.6)	367/12,331 (3.0)	31/2,807 (1.1)	24/1,138 (2.1)	55/3,945 (1.4)
**Turkey**	0/290 (0)	0/264 (0)	0/26 (0)	0/0 (0)	0/26 (0)
**United Kingdom**	10/331 (3.0)	1/195 (0.5)	2/72 (2.8)	7/64 (10.9)	9/136 (6.6)
**Ukraine**	1/177 (0.6)	1/145 (0.7)	0/30 (0)	0/2 (0)	0/32 (0)
**TOTAL**	**3,986/34,920 (11.4)**	**2,927/27,776 (10.5)**	**572/5,085 (11.2)**	**487/2,059 (23.7)**	**1,059/7,144 (14.8)**
